# Association Between Exposure to Smartphones and Tablets and Motor Development in Early Childhood: A Systematic Review

**DOI:** 10.1111/cch.70180

**Published:** 2025-11-15

**Authors:** Rinelly Pazinato Dutra, Yasmin Marques Castro, Veridiana Moran, Vicente Gabriel Wink Mattos, Paulo Victor Moura Rodrigues, Eliane Denise Araújo Bacil, Michael Pereira da Silva

**Affiliations:** ^1^ Faculty of Medicine Federal University of Rio Grande Rio Grande Brazil; ^2^ Department of Physical Education Central‐West State University Guarapuava Brazil

**Keywords:** child development, fine motor skills, gross motor skills, motor development, smartphones, tablets

## Abstract

**Background:**

This systematic review investigated the association between smartphone/tablet exposure and motor development in children aged 0–6 years.

**Methods:**

Observational studies in Portuguese, English or Spanish were included following the Participants, Exposure, Comparison, Outcomes and Study Design. A search conducted in November 2024 across seven databases identified 3228 records. After screening titles, abstracts and full texts, seven studies met the eligibility criteria, comprising sample sizes that ranged from 25 to 715 participants, for a combined total of 1339. Exposure was assessed via parental report, considering variables such as average daily time of use, frequency and age at first exposure, although definitions varied across studies. Methodological quality was assessed using the AXIS tool, and findings were synthesised qualitatively.

**Results:**

Findings were heterogeneous; one study reported negative associations with gross motor skills, two with fine motor skills and one with overall motor performance. Conversely, three studies indicated potential benefits for fine motor skills, and one found no significant associations. The relationship appears complex and may depend on the context, frequency and duration of use.

**Conclusions:**

The findings underscore the importance of guiding parents, educators and healthcare providers to balance smartphone/tablet exposure with motor‐enriching activities. Future longitudinal studies are needed to clarify causal pathways and the long‐term effects of these exposures (PROSPERO: CRD420251008664).

## 1 Introduction

Motor development is characterised by continuous changes in motor behaviour throughout life, resulting from the interaction between the environment, the task and the individual (Gallahue et al. 2013; Papalia and Martorell 2021). For motor skills to improve, children need to engage in activities appropriate to their developmental stage, as well as explore and interact with their environment, promoting experiences and developing essential competencies for subsequent life stages (Gallahue et al. 2013; Papalia and Martorell 2021; Payne and Isaacs 2012).

However, the advancement of the digital era has transformed these interactions, as the use of smartphones and tablets by children has grown significantly, occurring at increasingly younger ages (Ashton and Beattie 2019; Radesky and Christakis 2016; Trott et al. 2022). This increase, intensified by the COVID‐19 pandemic, raises concerns since screen interaction often competes with physical and social activities that are fundamental to motor development (Ashton and Beattie 2019; Radesky et al. 2015; Zeng et al. 2017).

Additionally, unlike television, mobile devices provide a more active interaction, allowing greater interactivity and on‐demand access to a vast range of content, which may result in distinct impacts on motor skill development (Ashton and Beattie 2019; Bernard et al. 2017; Panjeti‐Madan and Ranganathan 2023; Radesky et al. 2015). This active engagement, mediated by touch and gestures, can stimulate fine motor skills, such as hand–eye coordination. However, excessive use may reduce time spent on gross motor activities, which are essential for overall physical development (Panjeti‐Madan and Ranganathan 2023; Lin et al. 2017; Radesky et al. 2015).

Paediatric guidelines recommend avoiding screen exposure for children under 2 years old and limiting usage to a maximum of 1 h/day for those aged 2 to 5 years (Sociedade Brasileira de Pediatria 2024; World Health Organization 2019). These recommendations aim to minimise the potential negative impacts of screen time and ensure healthy development, given that early and excessive exposure is detrimental to various aspects of child health (Sociedade Brasileira de Pediatria 2024; World Health Organization 2019).

In light of these concerns, the number of studies examining the impacts of smartphone and tablet use on child development has been growing. However, previous research has indicated associations between prolonged exposure to passive screens (such as television) and delays in motor development (Felix et al. 2020; Lin et al. 2015; Madigan et al. 2019; Pagani et al. 2013; Poitras et al. 2017; Streegan et al. 2022), contemporary mobile devices introduce a distinct form of interaction whose effects remain underexplored. Although Arabiat et al. (2023) conducted a systematic review on interactive screens (including computers and video games), the exclusive impact of smartphones and tablets on motor development remains a gap in the literature, reinforcing the need for a comprehensive and focused synthesis on this topic.

Given this context, the present study aims to investigate the associations between exposure to smartphones and tablets, and the motor development of children aged 0 to 6 years through a systematic review of observational studies. By focusing on how these exposures occur in real‐life contexts, this review aims to summarise the available evidence on the association between these devices and children's motor development, providing insights for guiding parental practices and public policies.

## 2 Methods

### 2.1 Study Design

This systematic review of observational studies was conducted using the guidelines established in the Preferred Reporting Items for Systematic Reviews and Meta‐Analyses (PRISMA) statement (Page et al. 2021). This systematic review was prospectively registered in the International Prospective Register of Systematic Reviews (PROSPERO) under the number CRD420251008664. No deviations from the established protocol were made during the review process.

### 2.2 Research Question and PECOS Strategy

The following research question was developed for the systematic review: “Is exposure to smartphones and tablets associated with the motor development of children aged 0 to 6 years?” The question was formulated using the PECOS acronym—Participants, Exposure, Comparison, Outcomes and Study Design—as a guide to direct the searches, as presented in Box 1.

**BOX 1:** Search terms according to the PECOS strategy.

Strategy	Description	Descriptors
P	Children from 0 to 6 years old	Child, preschool child, paediatric, early childhood, infant, toddler
E	Smartphones and tablets	Smartphone, mobile phone, phone, tablet, interactive screen, mobile device, screen device
C	Children not exposed or less exposed to these devices	—
O	Motor development (gross and fine motor skills)	Motor development, motor performance, motor skills, gross motor skill, fine motor skill, motor coordination, motor behaviour
S	Observational studies: longitudinal (cohorts), cross‐sectional and case–control.	—

### 2.3 Eligibility Criteria

Studies were included analysing the association between smartphone and/or tablet use and the motor development of children aged 0 to 6 years, published in Portuguese, English or Spanish. Systematic, narrative or scoping reviews, letters to the editor, opinion articles, clinical trials, conference abstracts, purely descriptive analyses, studies addressing children outside the established age range (0–6 years), those including children with special health conditions, studies that analysed general screen use without specifying the devices (tablets and/or smartphones) in the analyses or that assessed interactive screens combined with other devices, such as video games, were excluded.

### 2.4 Databases and Search Strategy

In November 2024, searches were conducted in the PubMed, Embase, Web of Science, PsycINFO, LILACS, Scopus and SciELO databases. The search strategies, detailed in Supplementary Box S1, employed combinations of descriptors and Boolean operators within each platform's advanced search tools.

### 2.5 Article Selection Process

The articles retrieved from the databases were first exported to Zotero software, where duplicates were removed using the software's deduplication tools. Additional verification was performed manually, if necessary. Then they transferred to the Systematic Review Data Repository (SRDR+) tool for study management and selection for inclusion in this review.

The screening process consisted of two stages: (1) title and abstract screening and (2) full‐text evaluation of potentially eligible articles. Additionally, the reference lists of selected studies were examined to identify any additional relevant articles. Two independent reviewers conducted each stage and, in cases of disagreement, a third reviewer was consulted for the final decision regarding study inclusion.

### 2.6 Data Extraction

Data extraction was performed by two reviewers using a standardised form to collect information from the included studies. The extracted variables included author, year and country; study design; study objective; sample characteristics; instruments used to assess outcomes and exposures; the statistical analyses employed; key results, including prevalence, means and associations; and study limitations. After data extraction, the studies were organised in chronological order according to their year of publication.

### 2.7 Quality Assessment and Risk of Bias

The quality of the included studies was assessed using the Appraisal Tool for Cross‐Sectional Studies (AXIS) (Downes et al. 2016). This critical appraisal tool evaluates text quality and the risk of bias in cross‐sectional studies. This instrument consists of 20 items covering aspects such as study objectives clarity, justification, sample representativeness, validity and reliability of data collection methods, control of confounding factors, adequacy of statistical analysis, clarity in result presentation, discussion of limitations and transparency regarding ethical issues, funding and conflicts of interest. Each item had response options: “Yes,” “No,” or “Do not know”. Two reviewers independently conducted the evaluation, and a third reviewer resolved discrepancies. In this context, scores closer to 20 were considered indicative of higher methodological quality, while lower scores reflected a greater risk of bias, consistent with the internal consistency of the AXIS criteria.

### 2.8 Synthesis Methods and Effect Measures Used

The data were synthesised qualitatively, considering the heterogeneity of the studies regarding exposure measurement methods and outcomes. General study information (author, year, country and design), population characteristics (sample size, children's age) and instruments used to assess motor development and mobile device exposure were extracted and analysed. Smartphone and tablet exposure characteristics, such as usage prevalence, average exposure time, frequency and age at initial exposure, were also extracted and analysed.

This review focused on motor development variables, including outcomes associated with motor behaviour, motor skills and developmental milestones. While both cross‐sectional and longitudinal studies were eligible, all included studies were cross‐sectional, which meant that outcomes reflected motor performance at a single point in time. Nevertheless, the term “motor development” was applied broadly, in line with the theoretical construct used in the included studies and in the standardised assessment tools they employed. Data on motor development (gross and fine motor skills) included prevalence rates of delays in both domains, means/medians, standard deviations/interquartile ranges and standardised test scores.

The reported association measures in the included studies included Pearson or Spearman correlation coefficients, mean and/or median differences in motor tests between exposed and nonexposed groups, linear regressions and chi‐square tests. The data were organised in a table, maintaining the original presentation formats of the studies without additional transformations into other effect measures.

The studies were arranged chronologically and categorised according to the type of observed association: negative/harmful, positive/beneficial or not statistically significant. This classification was based on the reported statistical significance and the direction of the associations: results were considered beneficial when higher motor performance scores or better developmental outcomes were observed, harmful when lower scores or developmental delays were identified and as having no association when results were not statistically significant.

Meta‐analysis, subgroup analysis and meta‐regression were not conducted due to the high methodological variability among the included studies, mainly concerning exposure measurement methods, motor development assessment instruments and different statistical approaches. This hindered the standardisation of effect measures. Given these limitations, a qualitative synthesis was chosen, where results were described comparatively, highlighting the direction and magnitude of the reported associations. A certainty‐of‐evidence assessment (e.g., using the GRADE approach) was not conducted due to the substantial methodological and analytical heterogeneity of the included studies, which precluded the standardisation of outcomes and effect measures. Furthermore, none of the primary studies reported procedures for handling missing data.

## 3 Results

**FIGURE 1:**
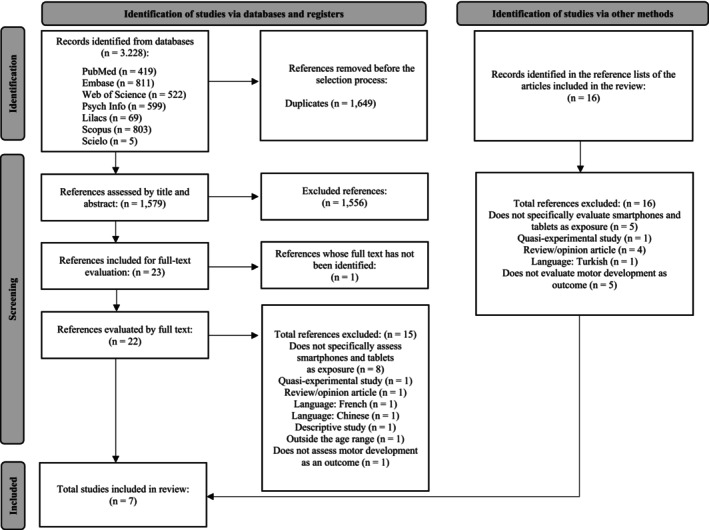
Flowchart for selecting articles for the systematic review on the association between the use of smartphones and tablets and children's motor development. Adapted from PRISMA (Page et al. 2021).

A total of 3228 articles were identified in the databases consulted, with 419 found in PubMed, 811 in Embase, 522 in Web of Science, 599 in PsycINFO, 69 in LILACS, 803 in Scopus and 5 in SciELO. After removing 1649 duplicates, 1579 articles proceeded to the title and abstract screening stage. The article selection process is illustrated in Figure 1.

In the initial screening, 1556 references were excluded for not meeting the established eligibility criteria, and one reference was excluded due to the unavailability of a full‐text version. Consequently, 22 articles were retained for full‐text review. After this review, 15 articles were excluded for not meeting the criteria (Supplementary Box S2), leaving 7 for inclusion in the present review. Additionally, a manual search was conducted in the reference lists of the included articles; however, no further studies were identified for inclusion.

### 3.1 Characteristics of the Included Studies

Seven cross‐sectional studies were included (Bedford et al. 2016; Chaibal and Chaiyakul 2022; Dadson et al. 2020; Lin 2019; Moon et al. 2019; Souto et al. 2019; Stamati et al. 2022), conducted in different countries, assessing the association between smartphone and tablet use and child motor development. Among the included studies, three were conducted in Asia (Chaibal and Chaiyakul 2022; Lin 2019; Moon et al. 2019), two in South America (Souto et al. 2019; Stamati et al. 2022), one in Europe (Bedford et al. 2016) and one in Oceania (Dadson et al. 2020). Regarding the publication period, the studies were published between 2016 and 2022. The first identified study was conducted in the United Kingdom in 2016 (Bedford et al. 2016), followed by three studies published in 2019—one in Taiwan (Lin 2019), one in South Korea (Moon et al. 2019) and one in Brazil (Souto et al. 2019). In 2020, a study was published in Australia (Dadson et al. 2020), while the most recent studies, from 2022, were conducted in Thailand (Chaibal and Chaiyakul 2022) and Argentina (Stamati et al. 2022).

The included studies involved children aged between 6 months and 7 years and their parents or caregivers, who provided information on smartphone and tablet use and various aspects of child development. Sample sizes ranged from 25 participants (Dadson et al. 2020) to 715 (Bedford et al. 2016), for a combined total of 1339 participants across the included studies. The studies analysed various aspects, including the frequency and duration of device use, the age at which exposure began and its effects on fine and gross motor skills. A detailed description of the studies included in this review can be found in Box 2, where they are listed chronologically by year of publication.

**BOX 2:** Presentation of the studies included in the review.

Author, year and country	Study design	Objective	Population and sample	Instruments and analysis	Main results
Bedford et al. (2016) United Kingdom	Cross‐sectional	To examine how the use of touchscreen devices varies from 6 to 36 months and to investigate the relationship between the age of first touchscreen use and the achievement of motor and language milestones in children aged 19 to 36 months.	715 children 6–36 months Average age: (19.5 ± 8.3) months Primary analysis carried out with 366 children aged 19 to 36 months.	**Parental report** **Touchscreen use:** Questionnaire adapted from other existing instruments. **Motor development:** Direct parental questions about key motor milestones (e.g., “At what age did he/she first”) **Statistical analysis:** Partial correlations controlling for age, gender, level of maternal education and corresponding early milestones.	75.2% of the children used touchscreen devices daily, with an average of 24.45 min/day. Mean age at first use of touchscreen devices: 13.13 ± 6.05 months Initial age at first use of touchscreen devices was positively associated with the achievement of fine motor milestones (stacking blocks: *r* = 0.16, *p* = 0.03), but there was no significant association for gross motor milestones (walking: *r* = 0.08, *p* = 0.21).
Lin (2019) Taiwan	Cross‐sectional	To investigate whether or not there were differences between preschool children who used tablets and those who did not use tablets in visual perception and fine motor skills.	72 children 4–6 years Average age: (61.9 ± 7.3) months	**Parental report** **Use of tablets:** Questionnaire if used and for how many days a week and for how long **Motor development:** Bruininks Oseretsky motor proficiency test, 2nd Ed (BOT‐2) **Statistical analysis:** *t* tests	**Prevalence of tablet use = 50% (*n* = 36)** **Fine motor assessment scores (mean ± SD)** Fine motor accuracy and tablet use = 18.67 ± 4.08 Fine motor accuracy and no tablet use = 21.78 ± 3.83 *p*‐value = 0, 001 Fine motor integration and use of tablets = 18.42 ± 3.57 Fine motor integration and no use of tablets = 20.75 ± 4.92 *p*‐value = 0.024 Manual dexterity and use of tablets = 17.22 ± 3.91 Manual dexterity and no use of tablets = 18.92 ± 3.21 *p*‐value = 0.048
Moon et al. (2019) South Korea	Cross‐sectional	To evaluate the relationships between factors related to the use of smart devices (smartphones, tablets and touchscreens), such as frequency of use, time of use and level of appropriate use and levels of development and language scores in young children.	117 children 3–5 years Average age: (4.5 ± 0.9) years	**Parental report** **Use of tablets and smartphones:** Parental questionnaire on the use of smart devices (frequency and time of use) **Motor development:** Korean developmental screening test **Statistical analysis:** Spearman's correlation	**Development scores:** **Gross motor skills:** −2 SD: 0.9% −2 to −1 SD: 21.1% −1 to +1 SD: 50.5% +1 SD: 27.5% **Fine motor skills:** −2 SD: 0.9% −2 to −1 SD: 11.0% −1 to +1 SD: 47.7% +1 SD: 40.4% **Frequency of device use** Do not use devices = 8.6% 1–4 times a week = 67.5% 5 days or more a week = 23.9% **Time spent using devices weekdays** <1 h/day = 70.1% ≥1 to <2 h/day = 25.6% ≥2 to <3 h/day = 3.4% >3 h/day = 0.9% **Time spent using devices weekends** <1 h/day = 60.1% ≥1 to <2 h/day = 31.6% ≥2 to <3 h/day = 6.0% >3 h/day = 1.9% **Correlation between frequency of device use and gross motor skills:** At 3 years: (ρ = −0.001 *p* = 0.994) At 4 years: (ρ = −0.047 *p* = 0.792) At 5 years: (ρ = 0.028 *p* = 0.869) **Correlation between time of use of devices and gross motor skills:** At 3 years: (ρ = −0.100 *p* = 0.556) At 4 years: (ρ = 0.176 *p* = 0.319) At 5 years: (ρ = −0.038 *p* = 0.819) **Correlation between frequency of device use and fine motor skills:** At 3 years: (ρ = 0.426 *p* = 0.009) At 4 years: (ρ = 0.061 *p* = 0.732) At 5 years: (ρ = 0.046 *p* = 0.786) **Correlation between time of device use and fine motor skills:** At 3 years: (ρ = −0.024 *p* = 0.889) At 4 years: (ρ = −0.233 *p* = 0.184) At 5 years: (ρ = −0.083 *p* = 0.621)
Souto et al. (2019) Brazil	Cross‐sectional	To assess whether the fine motor skills of young children who frequently use an interactive tablet differ from those who do not use this device	72 children 24–42 months Average age 36 months	**Parental report** **Tablet use:** Interactive media questionnaire assessed the use of interactive media (such as tablet and/or smartphone), including information on frequency and duration (in minutes) per day, and duration of exposure during the previous period (in months) **Motor development:** Bayley Scales of Infant Development, 3rd Ed (Bayley III) **Statistical analysis:** Chi‐square Mann–Whitney *U*‐test	**Prevalence of tablet use:** 20.2% **Average time of daily use:** 60 min **Median period the children used the tablet:** 9.5 months **Differences in median fine motor performance** Children using tablets = 12 (ranging from 9.0 to 19.0) Children not using tablets = 11.0 (ranging from 7 to 15) Difference between groups: *p*‐value = 0.013 The effect size of the difference was 0.66 (with a power of 70%; *p* < 0.05), indicating a moderate difference between the groups.
Dadson et al. (2020) Australia	Cross‐sectional	To explore the association between children's screen time, fine motor skills, manual manipulation (MMI), visual–motor integration (VMI), sensory processing (SP) and play skills.	25 children 4–7 years Average age: (6.2 ± 1.03) years	**Parental report** **Use of devices:** Daily record of screen time for 1 week **Motor development:** Bruininks Oseretsky test of motor proficiency, 2nd Ed (BOT‐2) Fine motor coordination subtests Test of in‐hand manipulation‐ revised (TIHM‐R) Beery Buktenica developmental test of visual–motor integration, Sixth Edition (Beery VMI) **Statistical Analysis:** Spearman correlation and linear regression (bootstrapping)	**Motor subscale averages** **VMI:** 105.3 ± 13.0 (average) **IHM:** 63.9 ± 14.7 (average) **Fine motor accuracy:** 27.0 ± 8.7 (average) **Fine motor integration:** 33.1 ± 6.0 (average) **Bilateral coordination:** 21.9 ± 2.4 (above average) **Prevalence of weekly use** Tablet (iPad) = 82.6% Smartphone (iPhone) = 21.7% **Average interactive screen time (min/week)** 168.9 ± 146.9 **Corr. interactive screen time and fine motor accuracy score** (ρ = −0.14; Value—*p* > 0.05) **Corr. interactive screen time and fine motor integration score** (ρ = −0.16; Value—*p* > 0.05) **Corr. interactive screen time and bilateral coordination score** (ρ = −0.11; Value—*p* > 0.05) **Corr. interactive screen time and IHM** (ρ = −0.46; Value—*p* < 0.05) **Corr. interactive screen time and VMI** (ρ = −0.52; Value—*p* < 0.05) **Linear reg. interactive screen time and fine motor skills** *F* = 4.55, *p* = 0.045, *R* ^2^ aj. = 0.139 (without bootstrap) With bootstrap (1000 samples) Value—*p* = 0.057 **Linear reg. interactive screen time and IHM** *F* = 5.28, *p* = 0.032, *R* ^2^ aj. = 0.169 (without bootstrap) With bootstrap (1000 samples) Value—*p* = 0.426 **Linear reg. total screen time and VMI** *F* = 17.39, *p* = 0.001, *R* ^2^ aj. = 0.427 (without bootstrap) With bootstrap (1000 samples) Value—*p* < 0.001
Chaibal and Chaiyakul (2022) Thailand	Cross‐sectional	Examining the correlation between the duration of smartphone and tablet use and child development	85 children 2–5 years Average age: (4.05 ± 0.91) years	**Parental report** **Use of devices:** Questionnaire, asking about time of use per day and time of day of use. **Motor development:** Denver Developmental Screening Test (Denver II) **Statistical analysis** Pearson's correlation *χ* ^2^ to analyse correlation between subcategories	**Average age of onset of smartphone/tablet use:** 2.77 ± 1.04 years **Average time of smartphone/tablet use:** 82.78 ± 62.82 min/day and 6.25 ± 1.42 days/week. **Prevalence of suspected developmental delay:** Gross motor: 2.4% and fine motor: 32.9% **Time spent using smartphones and tablets among children with:** Normal gross motor development: 81.32 ± 62.83 min/day Delayed gross motor development: 143.58 ± 15.15 min/day Normal fine motor development: 75.23 ± 48.61 min/day Delayed fine motor development: 98.17 ± 83.74 min/day **Association analysis (*χ*** ^**2**^ **)** **Association between children's smartphone/tablet use time and gross motor development:** (*χ* ^2^ = 6.657, *p* = 0.036) **Not significant for the fine motor domain:** (*χ* ^2^ = 0.632, *p* = 0.729)
Stamati et al. (2022) Argentina	Cross‐sectional	To describe the use of electronic media (i.e., TV, cell phone and tablet) and its association with motor and language milestones in the first years of life.	253 caregivers of children from 2 to 48 months Average age: (30.17 ± 10.82) months	**Parental report** **Use of screens:** Questionnaire on the type of touchscreen (TV, tablets and smartphones). Exposure time and purpose of use. **Motor development:** Questions on developmental milestones on a Likert scale with an indication of the age group at each milestone. **Statistical analysis:** Spearman's correlation	**Age at first exposure to cell phone:** 3.56 ± 1.62 months **Age at first exposure to tablet:** 5.15 ± 1.41 months **Average time using cell phone:** 1.72 ± 0.82 h/day **Average time using tablet:** 1.35 ± 0.74 h/day **Average motor development milestones:** 3.73 ± 0.49 **Correlation analysis:** **Age at first use of cell phone and motor development:** (ρ = −0.217; *p*‐value < 0.01) **Age at first use of tablet and motor development:** (ρ = −0.060; *p*‐value > 0.05) **Usage time of cell phone and motor development:** (ρ = 0.011; *p*‐value > 0.05) **Usage time of tablet and motor development:** (ρ = −0.026; *p*‐value > 0.05)

### 3.2 Assessment Methods (Outcomes and Exposures)

All seven studies used parental reporting as the data collection method to obtain information about the children. Of these, five employed validated instruments to assess motor development (Chaibal and Chaiyakul 2022; Lin 2019; Moon et al. 2019; Souto et al. 2019; Dadson et al. 2020). Bedford et al. (2016) assessed key motor milestones through direct questions about the age at which each milestone was achieved, and Stamati et al. (2022), using a similar approach, applied a Likert scale with age ranges for each developmental milestone (Box 2). All studies used self‐developed instruments to evaluate exposure to smartphones and tablets, with varying measurement approaches concerning the types of devices and exposure time. The studies by Lin (2019) and Souto et al. (2019) specifically assessed tablet use, while the study by Stamati et al. (2022) analysed tablet and smartphone use separately. On the other hand, the studies by Bedford et al. (2016), Chaibal and Chaiyakul (2022), Dadson et al. (2020) and Moon et al. (2019) evaluated tablet and smartphone use jointly.

In terms of measurement approach, six studies used structured questionnaires to gather information on the frequency and daily exposure time to the devices (Bedford et al. 2016; Chaibal and Chaiyakul 2022; Lin 2019; Moon et al. 2019; Souto et al. 2019; Stamati et al. 2022). In contrast, only one study used daily logs completed by parents over 1 week to more accurately measure device exposure time (Dadson et al. 2020).

### 3.3 Device Exposure Patterns (Prevalence, Frequency, Duration, Onset Age)

Regarding daily device use, Bedford et al. (2016) found a prevalence of 75.2% for smartphones/tablets, while Lin (2019) and Souto et al. (2019) reported prevalence of 50% and 20.2%, respectively, for tablet use. Dadson et al. (2020) observed that 82.6% of children used tablets weekly, while 21.7% used smartphones. The mean age of first exposure ranged from 13.13 ± 6.05 months (Bedford et al. 2016) to 2.77 ± 1.04 years (Chaibal and Chaiyakul 2022) for smartphones/tablets, and even earlier in the study by Stamati et al. (2022), which reported 3.56 ± 1.62 months for cell phones and 5.15 ± 1.41 months for tablets.

Usage frequency also varied across studies. Moon et al. (2019) found that 67.5% of children used devices between one and four times per week, while 23.9% used them five or more days per week. Chaibal and Chaiyakul (2022) reported a mean frequency of 6.25 ± 1.42 days per week. The average daily use time for both devices was 24.45 min/day in the study by Bedford et al. (2016), 60 min/day in Souto et al. (2019) and 82.78 min/day in Chaibal and Chaiyakul (2022). In the study by Stamati et al. (2022), children spent an average of 1.72 ± 0.82 h/day on cell phones and 1.35 ± 0.74 h/day on tablets. Meanwhile, Dadson et al. (2020) assessed average weekly usage and found that children were exposed to smartphones and/or tablets for an average of 168.9 min per week. Finally, Moon et al. (2019), when analysing usage time across both devices, observed that on weekdays, 70.1% of children used devices for less than 1 h/day, 25.6% used them for 1 to 2 h/day, 3.4% for 2 to 3 h/day and 0.9% for more than 3 h/day. On weekends, usage was higher, with 60.1% using them for less than 1 h/day, 31.6% for 1 to 2 h, 6% for 2 to 3 h/day and 1.9% for more than 3 h/day.

### 3.4 Motor Development Results (Fine and Gross Motor Skills Scores, Developmental Milestones)

Regarding motor development assessments, Lin (2019) evaluated fine motor skills and reported mean scores for fine motor precision (18.67 ± 4.08), fine motor integration (18.42 ± 3.57) and manual dexterity (17.22 ± 3.91) in the children assessed. In the study by Moon et al. (2019), 27.5% of children scored above average (+1 SD or more), 50.5% were within the average range (−1 to +1 SD), 21.1% scored between −2 and −1 SD (below average performance) and 0.9% scored below −2 standard deviations (significant delay) for gross motor skills. Regarding fine motor skills, 40.4% of children scored +1 SD or more, 47.7% were in the average range (−1 to +1 SD), 11.0% scored between −2 and −1 SD and 0.9% scored below −2 SD. In the motor subtests conducted in the study by Dadson et al. (2020), the mean scores obtained in motor skills tests were VMI (105.3 ± 13.0), IHM (63.9 ± 14.7), fine motor precision (27.0 ± 8.7), fine motor integration (33.1 ± 6.0), all classified as average, and bilateral coordination (21.9 ± 2.4), classified as above average. Chaibal and Chaiyakul (2022) identified that 2.4% of the children showed a suspected delay in gross motor development, while 32.9% showed a suspected delay in fine motor development. Additionally, in the study by Stamati et al. (2022), which assessed motor milestones through parental reports, the average score was 3.73 ± 0.49, according to the Likert scale used.

### 3.5 Associations Between Device Exposure and Motor Development

Specifically, regarding the association analyses (Box 3), while cross‐sectional studies do not allow for conclusions about causal directionality, the included studies explored the research question from different perspectives. Some studies considered motor development as the outcome, investigating whether children exposed to smartphones and tablets exhibited better or poorer motor performance. Others treated device exposure as the outcome, evaluating whether children with typical or delayed motor development were more or less exposed to these devices. In both scenarios, the analyses only assessed the association between smartphone and tablet exposure and children's motor development without adjusting for covariates. It is also important to note that only one study (Bedford et al. 2016) adjusted for potential confounders, such as age, sex and mother's educational level.

**BOX 3:** Summary of the associations between exposure to smartphones and tablets and children's motor development in the included studies.

Study	Gross motor skills	Fine motor skills
Bedford et al. (2016)	➖	✔
Lin (2019)	➖	❌
Moon et al. (2019)	➖	✔
Souto et al. (2019)	➖	✔
Dadson et al. (2020)	**NE**	❌
Chaibal and Chaiyakul (2022)	❌	➖
Stamati et al. (2022)	❌ (Motor development)	

**Caption:** ✔️ = Positive/beneficial association; ❌ = negative/harmful association; ➖ = no association; **NE** = not evaluated.

Four studies (Chaibal and Chaiyakul 2022; Dadson et al. 2020; Lin 2019; Stamati et al. 2022) identified a negative association between smartphone and tablet use and child motor development, indicating a potentially harmful impact. Lin (2019) observed that children who used tablets had lower fine motor precision (18.67 ± 4.08) compared to those who did not use them (21.78 ± 3.83; *p* = 0.001), with the same pattern found for fine motor integration (18.42 ± 3.57 vs. 20.75 ± 4.92; *p* = 0.024) and manual dexterity (17.22 ± 3.91 vs. 18.92 ± 3.21; *p* = 0.048). Dadson et al. (2020) found that device exposure time was negatively correlated with both visual–motor integration (VMI) (ρ = −0.52; *p* < 0.05) and manual manipulation (IHM) (ρ = −0.46; *p* < 0.05). Moreover, in the linear regression analysis, the authors indicated that longer exposure time was associated with poorer performance in VMI (*F* = 17.39; *p* = 0.001; adj. *R* ^2^ = 0.427), IHM (*F* = 5.28; *p* = 0.032; adj. *R* ^2^ = 0.169) and fine motor skills (*F* = 4.55; *p* = 0.045; adj. *R* ^2^ = 0.139). Chaibal and Chaiyakul (2022) identified that children with gross motor development delay had a higher average daily exposure time to smartphones and tablets (143.58 ± 15.15 min/day) compared to those with typical development (81.32 ± 62.83 min/day), and this association was statistically significant (*χ* ^2^ = 6.657; *p* = 0.036). Finally, Stamati et al. (2022) found that the age at which cell phone use began was negatively correlated with motor development (ρ = −0.217; *p* < 0.01), suggesting that children exposed to these devices early showed poorer motor performance.

On the other hand, three studies (Bedford et al. 2016; Moon et al. 2019; Souto et al. 2019) went in the opposite direction and identified positive associations, i.e., potential benefits for child motor development, particularly fine motor skills. Bedford et al. (2016) found a positive association between the age at which smartphone use began and the achievement of fine motor milestones, such as stacking blocks (*r* = 0.16; *p* = 0.03). Souto et al. (2019) reported differences in the medians of fine motor performance between children who used tablets (Md = 12.0) and those who did not (Md = 11.0), and this difference was statistically significant (*p* = 0.013). Moon et al. (2019) indicated that the frequency of smartphone and tablet use was positively correlated with the development of fine motor skills at 3 years of age (ρ = 0.426; *p* = 0.009); however, no associations were observed for other age groups, suggesting that the impact may depend on the child's age at the time of exposure.

Although all studies identified some positive or negative association between smartphone and tablet use and child motor development, some specific analyses did not find statistically significant associations in certain motor domains, suggesting that the relationship may depend on even more complex factors. Although Bedford et al. (2016) found positive associations with fine motor milestones, they did not identify an association between device use and the achievement of gross motor milestones, such as walking (*r* = 0.08; *p* = 0.21). Similarly, Moon et al. (2019), although they reported positive results for fine motor skills, did not find significant correlations between time or frequency of device use and gross motor development in any age groups analysed.

Likewise, but in different domains, Chaibal and Chaiyakul (2022) observed that although there was an association with gross motor skills, screen time was not significantly related to fine motor development (*χ* ^2^ = 0.632; *p* = 0.729). Dadson et al. (2020) found no significant correlation between exposure time and the subtests of fine motor precision (ρ = −0.14; *p* > 0.05), fine motor integration (ρ = −0.16; *p* > 0.05) or bilateral coordination (ρ = −0.11; *p* > 0.05). Finally, Stamati et al. (2022) also found no associations between the age at which tablet use began and motor development (ρ = −0.060; *p* > 0.05), or between the usage time of cell phones and tablets and child motor development (ρ = 0.011; *p* > 0.05 and ρ = −0.026; *p* > 0.05, respectively).

### 3.6 Quality Analysis of Studies

The quality assessment (Box 4) identified the strengths and weaknesses and the methodological limitations of the studies included in the present review. All the studies scored were classified as having satisfactory methodological quality, with scores ranging from 14 (Souto et al. 2019) to 16 (Bedford et al. 2016) on the AXIS tool. All studies presented clear objectives and employed appropriate designs to address their research questions. However, none of the studies justified their sample size or reported the nonresponse rate, and all relied on nonprobabilistic sampling methods, which may limit the representativeness of their findings. Regarding data sources, motor development outcomes were adequately measured using validated instruments. However, all included studies assessed exposure to devices solely through parental reporting. None of the articles reported response rates or categorised nonrespondents, which may indicate selection bias, particularly in combination with the sampling methods.

**BOX 4:** Quality assessment and risk of bias of included studies.

Study	Q1	Q2	Q3	Q4	Q5	Q6	Q7	Q8	Q9	Q10	Q11	Q12	Q13	Q14	Q15	Q16	Q17	Q18	Q19	Q20
Bedford et al. (2016)	Y	Y	Y	Y	N	N	N	Y	Y	Y	Y	Y	N	N	Y	Y	Y	Y	N	Y
Lin (2019)	Y	Y	N	Y	N	N	N	Y	Y	Y	Y	Y	N	N	Y	Y	Y	Y	N	Y
Moon et al. (2019)	Y	Y	N	Y	N	N	N	Y	Y	Y	Y	Y	N	N	Y	Y	Y	Y	N	Y
Souto et al. (2019)	Y	Y	N	Y	N	N	N	Y	Y	Y	Y	Y	N	N	Y	Y	Y	Y	D.K	Y
Dadson et al. (2020)	Y	Y	N	Y	N	N	N	Y	Y	Y	Y	Y	N	N	Y	Y	Y	Y	N	Y
Chaibal and Chaiyakul (2022)	Y	Y	N	Y	N	N	N	Y	Y	Y	Y	Y	N	N	Y	Y	Y	Y	N	Y
Stamati et al. (2022)	Y	Y	N	Y	N	N	N	Y	Y	Y	Y	Y	N	N	Y	Y	Y	Y	N	Y

Constructed by the authors, based on 20 questions from the Appraisal Tool for Cross‐Sectional Studies (AXIS) (Downes et al. 2016). Legend: Y, yes; N, no; D.K, do not know.

Despite these limitations, all studies adequately described their methods and statistical analyses and presented results consistent with the methodologies employed. Finally, all articles discussed the study's limitations, but only six of the seven studies disclosed funding sources and/or conflicts of interest. The exception was Souto et al. (2019), who provided no such information in the article. Overall, the studies demonstrated satisfactory methodological quality, although specific issues must be considered when interpreting and summarising the findings.

## 4 Discussion

This systematic review explored the associations between exposure to smartphones and tablets and motor development in early childhood. Despite the increasing number of studies on this topic, the literature remains marked by inconsistencies and methodological heterogeneity, making it difficult to draw definitive conclusions. Our findings point to a complex and still inconclusive relationship between the use of these devices and children's motor development, with variable effects depending on the motor domain assessed. Negative associations were observed with gross motor skills, while fine motor skills presented divergent results, with some studies indicating impairments and others suggesting benefits. This complexity highlights the need for a critical analysis of the findings, considering the underlying mechanisms and the limitations of existing studies.

Regarding gross motor skills, most studies did not find statistically significant associations (Bedford et al. 2016; Lin 2019; Moon et al. 2019; Souto et al. 2019). However, in the analyses that did identify associations, it was observed that children with a higher average daily exposure time to devices showed delays in gross motor development, with considerable differences between children with and without delays (Chaibal and Chaiyakul 2022), while those who began using cell phones at an earlier age demonstrated poorer overall motor performance, although the correlations were weak (Stamati et al. 2022). Part of the explanation for these findings lies in replacing physical activities and outdoor play with smartphones and tablets, reducing opportunities for improving gross motor skills. Activities such as running, jumping, climbing and playing in open spaces are essential for muscle strengthening, balance and coordination (Ashton and Beattie 2019; Gallahue et al. 2013; Payne and Isaacs 2012; Sociedade Brasileira de Pediatria 2024).

Reviews on general screen use have associated prolonged exposure with delays in motor development, especially in gross motor skills (Madigan et al. 2019; Poitras et al. 2017). From the perspective of interactive devices, Radesky et al. (2015) highlight that these resources compete with essential motor activities that are fundamental for developing skills and competencies that promote autonomy. When the time devoted to such activities is reduced in favour of electronic device use, children may miss important opportunities to practice and refine fundamental movements, which may explain the delays observed in some studies (Ashton and Beattie 2019; Radesky et al. 2015; Zeng et al. 2017). Furthermore, interaction with the physical environment and sensory exploration are fundamental components of both motor and cognitive development, and their absence may have broader implications for later developmental stages (Bento and Dias 2017; Gallahue et al. 2013; Payne and Isaacs 2012; Varadarajan et al. 2021).

Regarding fine motor skills, the studies included in this review presented conflicting results. On the one hand, early exposure and more frequent use of devices may be associated with better performance in fine motor skills such as pointing and manipulating small objects, although this association was not observed with screen time nor sustained in older children (Bedford et al. 2016; Moon et al. 2019; Souto et al. 2019). This hypothesis is supported by the idea that the repetition of fine gestures during screen interaction could stimulate motor refinement (Bedford et al. 2016; Moon et al. 2019). However, as these are cross‐sectional studies limited to mean difference and correlation analyses, with some showing moderate effects in younger children and others reporting weak associations, caution is needed when interpreting the results. The lack of consistency in older age groups, the absence of a relationship with screen time and the lack of control for other sociodemographic and contextual variables suggest that potential benefits may be limited.

On the other hand, some studies (Dadson et al. 2020; Lin 2019) found weak to moderate negative associations between prolonged device use and fine motor skills, particularly in tasks requiring precision and visual–motor integration, suggesting that repetitive screen use may not be sufficient to stimulate the development of these skills adequately. Excessive screen exposure may limit opportunities for activities essential to fine motor development, such as playing with blocks, puzzle games, drawing and writing, which require muscle strength and motor control (Flewitt et al. 2015; Lauricella et al. 2015). Additionally, experimental evidence reinforces this concern, showing that children not exposed to tablets demonstrated more significant gains in fine motor precision and integration compared to those who were regularly exposed, as touchscreen actions such as swiping and tapping are less complex and require less strength than traditional activities such as writing or drawing (Lin et al. 2017). However, the clinical trial by Axford et al. (2018) indicated that moderate use of specific iPad applications (30 min per day) could improve motor coordination, while excessive use (> 30 min per day) resulted in negative effects. These findings underscore the need to investigate the presence or absence of exposure and the context of use, duration of exposure, quality of interaction and balance with diverse motor experiences to understand the impacts on motor development better.

A systematic review conducted by Arabiat et al. (2023) observed that using interactive digital technology (computers, tablets, video games, smartphones) may not lead to significant gains or may even be negatively associated with fine motor development. Although the review by Arabiat et al. (2023) makes relevant contributions by addressing the impact of interactive digital technologies on child development, there are important differences compared to the present study. While Arabiat et al. (2023) analysed various devices, this review focuses exclusively on the effects of smartphones and tablets, which are increasingly accessible and integrated into children's daily lives. Furthermore, our review included only observational studies based on natural usage contexts, whereas Arabiat et al. (2023) also analysed clinical trials and experimental interventions. Another relevant aspect is that our analysis specifically addressed associations with the domains of fine and gross motor skills in a detailed and qualitative manner. At the same time, the review by Arabiat et al. (2023) had a broader and more general scope. These differences reinforce the specificity and contribution of the present study to understanding the possible impacts of smartphone and tablet use on the motor development of young children.

Regarding the main limitations, the methodological heterogeneity of the included studies was a significant challenge for this review. The variation in motor development assessment instruments and the methods used to measure exposure made it difficult to compare results directly and prevented the performance of a meta‐analysis. Moreover, age ranges varied widely, which may have influenced the findings since motor development occurs in distinct phases and at different rates throughout early childhood (Gallahue et al. 2013). Another significant point concerns the type of study design. This review intentionally focused on observational studies, aiming to capture children's real‐world exposure to smartphones and tablets in everyday contexts, rather than controlled experimental interventions. However, among the studies that met the eligibility criteria, all studies were cross‐sectional, thus compromising the analysis of these devices' long‐term effects and precluding causal inference regarding their associations with children's motor development variables. In addition, most studies did not adjust their analyses for potential confounders, which further limits the interpretation of the associations reported.

The small sample size was a limitation in most of the studies (Chaibal and Chaiyakul 2022; Dadson et al. 2020; Lin 2019; Moon et al. 2019; Souto et al. 2019), as was the sampling process, which did not ensure a representative sample (Bedford et al. 2016; Dadson et al. 2020; Stamati et al. 2022). Additionally, the studies reported, as a limitation, the lack of analysis regarding the characteristics of device exposure, indicating that aspects such as the usage context, content consumed and parental control should be considered in future analyses (Bedford et al. 2016; Chaibal and Chaiyakul 2022; Dadson et al. 2020; Lin 2019). Lastly, although parental reporting was widely used as a data collection method, it was identified as a limitation due to the potential for recall bias. However, this approach was adopted due to the difficulty of obtaining such information through other means. Strategies to mitigate these biases should be implemented in future studies to ensure the reliability of the data collected.

Despite the aforementioned limitations, this systematic review provides a comprehensive synthesis of the available evidence on the relationship between smartphone and tablet use and children's motor development, highlighting the need for more standardised and longitudinally designed approaches. The findings indicate that the amount of time children use these devices may influence different aspects of motor development, with potentially positive or negative impacts depending on the child's age and the context of exposure. However, it is important to emphasise that the level of available evidence is limited since most studies adopted cross‐sectional designs with small samples and limited analytical control. These methodological limitations compromise the ability to infer causality and generalise the results and hinder the identification of consistent patterns, resulting in fragile conclusions.

The review's main findings indicate that the relationship between smartphone and tablet use and children's motor development is complex and still inconclusive, varying according to the type of exposure analysed, the child's age and the outcomes assessed. While some studies suggest possible benefits for fine motor skills, others point to negative effects on visual–motor integration and gross motor skills. Moreover, despite most studies reporting various associations, a substantial portion failed to demonstrate statistically significant findings across certain motor domains in the children evaluated. Compounding these issues, most of the reported associations were of small to moderate magnitude, suggesting that even where statistically significant, the practical or clinical impact of these effects might be limited and potentially not warrant specific interventions. Furthermore, the absence of statistical adjustments for other covariates constituted a major drawback in the majority of the included studies. Key confounding variables, such as socioeconomic status, parental education, pre‐existing developmental conditions or even other forms of screen time (e.g., educational apps vs. passive viewing), were often not accounted for. This omission makes it difficult to ascertain whether the observed effects are genuinely attributable to smartphone and tablet use or are merely spurious correlations driven by unmeasured factors.

These aspects collectively raise profound questions about clinical, practical and contextual relevance, as other, more influential variables, such as overall physical activity levels, genetic predispositions, the quality of the home learning environment or direct caregiver interaction, might mask or even overshadow the minor effects of smartphones and tablets on motor development variables. Nevertheless, given the developmental sensitivity of early childhood, a period characterised by rapid brain maturation and the foundational acquisition of motor skills essential for later development, these associations warrant continued and rigorous attention.

Despite these inconsistencies, it remains essential to support parents and caregivers in balancing mobile device use with experiences that promote motor development, such as outdoor play and social interaction. Translating these findings into practical guidance involves considering not only screen time but also the child's age, the context of use and the diversity of physical activities. This approach reinforces existing paediatric recommendations and provides important support for public policies aimed at promoting responsible and developmentally appropriate use of smartphones and tablets in early childhood.

Future research should improve statistical models, including appropriate adjustment for confounding variables, incorporate details about the content consumed and provide longitudinal data to better understand the association between smartphone/tablet use and children's motor development. Additionally, it is essential to investigate how parental supervision, the age of onset and duration of exposure and specific age ranges may modulate these effects, offering a stronger foundation for scientific understanding and evidence‐based recommendations.

## Author Contributions

**Rinelly Pazinato Dutra:** conceptualization, data curation, formal analysis, investigation, methodology, project administration, resources, software, supervision, validation, visualization, writing – original draft, writing – review and editing. **Yasmin Marques Castro:** investigation, methodology, writing – original draft, writing – review and editing. **Veridiana Moran:** investigation, methodology, writing – original draft, writing – review and editing. **Vicente Gabriel Wink Mattos:** investigation, methodology, writing – original draft, writing – review and editing. **Paulo Victor Moura Rodrigues:** investigation, methodology, writing – original draft, writing – review and editing. **Eliane Denise Araújo Bacil:** writing – original draft, writing – review and editing. **Michael Pereira da Silva:** conceptualization, formal analysis, investigation, methodology, writing – original draft, writing – review and editing.

## Conflicts of Interest

The authors declare no conflicts of interest.

## Supporting information


**Box S1** Search strategies in their respective databases.

**Box S2** Excluded studies and reasons for exclusion.

## Data Availability

The data analysed during this systematic review are publicly available through the original published studies. A complete list of all studies included in the review is available from the corresponding author upon request.
