# Supplementary material for: Association Between Exposure to Smartphones and Tablets and Motor Development in Early Childhood: A Systematic Review
Source: Child Care Health Dev. 2025 Nov 15;51(6):e70180. doi: 10.1111/cch.70180 (PMC12619079; doi:10.1111/cch.70180)
Supplement: Supplementary file 1 — Box S1 Search strategies in their respective databases. [file CCH-51-e70180-s001.docx]

**Supplementary Box 1:** Search strategies in their respective databases

| **Base Data** | **Search strategy** | **N** | **Date** |
| --- | --- | --- | --- |
| **PubMed** | *("child*"[All Fields] OR "preschool child"[All Fields] OR "pediatric"[All Fields] OR "early childhood"[All Fields] OR "infant"[All Fields] OR "toddler"[All Fields]) AND ("smartphon*"[All Fields] OR "mobile phon*"[All Fields] OR "phon*"[All Fields] OR "tablet*"[All Fields] OR "interactive screen*"[All Fields] OR "mobile device"[All Fields] OR "screen device"[All Fields]) AND ("motor development"[All Fields] OR "motor performance"[All Fields] OR "motor skills"[All Fields] OR "gross motor skill"[All Fields] OR "fine motor skill"[All Fields] OR "motor coordination"[All Fields] OR "motor behav*"[All Fields])* | **419** | 25/11/2024 |
| **Embase** | *('child*' OR 'preschool child'/exp OR 'pediatric'/exp OR 'early childhood'/exp OR 'infant'/exp OR 'toddler'/exp) AND ('smartphon*' OR 'mobile phon*' OR 'phon*' OR 'tablet*' OR 'interactive screen*' OR 'mobile device*' OR 'screen device*') AND ('motor development'/exp OR 'motor performance'/exp OR 'motor skills' OR 'gross motor skill'/exp OR 'fine motor skill'/exp OR 'motor coordination' OR 'motor behav*')* | **811** | 25/11/2024 |
| **Web of Science** | *(ALL=child* OR ALL="preschool child" OR ALL=pediatric OR ALL="early childhood" OR ALL=infant OR ALL=toddler) AND (ALL=smartphon* OR ALL="mobile phon*" OR ALL=phon* OR ALL=tablet* OR ALL="interactive screen*" OR ALL="mobile device*" OR ALL="screen device*") AND (ALL="motor development" OR ALL="motor performance" OR ALL="motor skills" OR ALL="gross motor skill" OR ALL="fine motor skill" OR ALL="motor coordination" OR ALL="motor behav*")* | **522** | 25/11/2024 |
| **PsycINFO** | *(Any Field: child* OR Any Field: "preschool child" OR Any Field: pediatric OR Any Field: "early childhood" OR Any Field: infant OR Any Field: toddler) AND (Any Field: smartphon* OR Any Field: "mobile phon*" OR Any Field: phon* OR Any Field: tablet* OR Any Field: "interactive screen*" OR Any Field: "mobile device*" OR Any Field: "screen device*") AND (Any Field: "motor development" OR Any Field: "motor performance" OR Any Field: "motor skills" OR Any Field: "gross motor skill" OR Any Field: "fine motor skill" OR Any Field: "motor coordination" OR Any Field: "motor behav*)* | **599** | 25/11/2024 |
| **LILACS** | *(child* OR "preschool child" OR pediatric OR "early childhood" OR infant OR toddler) AND (smartphon* OR "mobile phon*" OR phon* OR tablet* OR "interactive screen*" OR "mobile device*" OR "screen device*") AND ("motor development" OR "motor performance" OR "motor skills" OR "gross motor skill" OR "fine motor skill" OR "motor coordination" OR "motor behav*")* | **69** | 25/11/2024 |
| **Scopus** | *( TITLE-ABS-KEY ( child* ) OR TITLE-ABS-KEY ( "preschool child" ) OR TITLE-ABS-KEY ( pediatric ) OR TITLE-ABS-KEY ( "early childhood" ) OR TITLE-ABS-KEY ( infant ) OR TITLE-ABS-KEY ( toddler ) AND TITLE-ABS-KEY ( smartphon* ) OR TITLE-ABS-KEY ( "mobile phon*" ) OR TITLE-ABS-KEY ( phon* ) OR TITLE-ABS-KEY ( tablet* ) OR TITLE-ABS-KEY ( "interactive screen*" ) OR TITLE-ABS-KEY ( "mobile device*" ) OR TITLE-ABS-KEY ( "screen device*" ) AND TITLE-ABS-KEY ( "motor development" ) OR TITLE-ABS-KEY ( "motor performance" ) OR TITLE-ABS-KEY ( "motor skills" ) OR TITLE-ABS-KEY ( "gross motor skill" ) OR TITLE-ABS-KEY ( "fine motor skill" ) OR TITLE-ABS-KEY ( "motor coordination" ) OR TITLE-ABS-KEY ( "motor behav*" ) )* | **803** | 25/11/2024 |
| **SciELO** | *(child* OR "preschool child" OR pediatric OR "early childhood" OR infant OR toddler) AND (smartphon* OR "mobile phon*" OR phon* OR tablet* OR "interactive screen*" OR "mobile device*" OR "screen device*") AND ("motor development" OR "motor performance" OR "motor skills" OR "gross motor skill" OR "fine motor skill" OR "motor coordination" OR "motor behav*")* | **5** | 25/11/2024 |
