# Supplementary material for: Association Between Exposure to Smartphones and Tablets and Motor Development in Early Childhood: A Systematic Review
Source: Child Care Health Dev. 2025 Nov 15;51(6):e70180. doi: 10.1111/cch.70180 (PMC12619079; doi:10.1111/cch.70180)
Supplement: Supplementary file 2 — Box S2 Excluded studies and reasons for exclusion. [file CCH-51-e70180-s002.docx]

**Supplementary Box 2:** Excluded studies and reasons for exclusion

| **Authors** | **Year** | **Article title** | **DOI** | **Reason for exclusion** |
| --- | --- | --- | --- | --- |
| Tomopoulos *et al.* | 2010 | Infant media exposure and toddler development | 10.1001/archpediatrics.2010.235 | Outcome: Not assessed Motor development |
| Brown | 2011 | Media use by children younger than 2 years: policy statement of the AAP | 10.1542/peds.2011-1753 | Design: Review / Opinion article |
| Christakis | 2014 | Interactive media use at younger than the age of 2 years: time to rethink the American Academy of Pediatrics Guideline? | 10.1001/jamapediatrics.2013.5081 | Design: Review / Opinion article |
| Ahearne *et al.* | 2015 | Touch screen technology usage in toddlers | 10.1136/archdischild-2015-309278 | Outcome: Not assessed Motor development |
| Cristia, *et al.* | 2015 | Parental reports on touch screen use in Early childhood | 10.1371/journal.pone.0128338 | Outcome: Not assessed Motor development |
| Kabali *et al.* | 2015 | Exposure and use of mobile media devices by young children | 10.1542/peds.2015-2151 | Outcome: Not assessed Motor development |
| Price *et al.* | 2015 | The role of iPads in pre-school children's mark making development | 10.1016/j.compedu.2015.04.003 | Design: Quasi-experimental |
| Xu, *et al.* | 2016 | A 5-year longitudinal analysis of modifiable predictors for outdoor play and screen-time of 2- to 5-year-olds | 10.1186/s12966-016-0422-6 | Exposure: Only evaluates screen time in general |
| Li, *et al.* | 2017 | Effect of Touch Screen Tablet Use on Fine Motor Development of Young Children | 10.1080/01942638.2016.1255290 | Design: Quasi-experimental |
| Cadoret *et al.* | 2018 | Relationship between screen-time and motor proficiency in children: A longitudinal study. | 10.1080/03004430.2016.1211123 | Exposure: Only evaluates screen time in general |
| Mustafaoglu *et al.* | 2018 | The negative effects of digital technology usage on children’s development and health. | 10.15805/addicta.2018.5.2.0051 | Language: Turkish |
| Madigan *et al.* | 2019 | Association between screen time and children’s performance on a developmental screening test | 10.1001/jamapediatrics.2018.5056 | Exposure: Only evaluates screen time in general |
| Webster *et al.* | 2019 | Fundamental motor skills, screen-time, and physical activity in pre-schoolers | 10.1016/j.jshs.2018.11.006 | Exposure: Only evaluates screen time in general |
| Félix *et al.* | 2020 | Excessive screen media use in preschoolers is associated with poor motor skills | 10.1089/cyber.2019.0238 | Exposure: Only evaluates screen time in general |
| Marcelli *et al.* | 2020 | Early and excessive exposure to screens (EEES): A new syndrome | 10.3917/dev.202.0119 | Language: French |
| Radesky *et al.* | 2020 | Young children’s use of smartphones and tablets. | 10.1542/peds.2019-3518 | Outcome: Not assessed Motor development |
| Chauan *et al.* | 2021 | Exposure to Smartphone and Screen media in Children and Adolescents and COVID-19 pandemic | 10.1177/0973134220210215 | Design: Review / Opinion article |
| Hossain *et al.* | 2021 | International study of 24-h movement behaviors of early years (SUNRISE): a pilot study from Bangladesh | 10.1186/s40814-021-00912-1 | Exposure: Only evaluates screen time in general |
| McArthur *et al.* | 2021 | Screen time and developmental and behavioral outcomes for preschool children | 10.1038/s41390-021-01572-w | Exposure: Only evaluates screen time in general |
| Rocha *et al.* | 2021 | Screen time and early childhood development in Ceará, Brazil: a population-based study | 10.1186/s12889-021-12136-2 | Exposure: Only evaluates screen time in general |
| Suggate *et al.* | 2021 | Children’s sensorimotor development in relation to screen-media usage: A two-year longitudinal study | 10.1016/j.appdev.2021.101279 | Exposure: Includes other devices in the analysis of other screens. |
| Yaday *et al.* | 2021 | Child-smartphone interaction: Relevance and positive and negative implications. | 10.1007/s10209-021-00807-1 | Design: Review / Opinion article |
| García *et al.* | 2022 | Screen use among toddlers and preschool children | 10.5546/aap.2022.eng.340 | Design: Review / Opinion article |
| Mulé *et al.* | 2022 | Correlation between Language Development and Motor Skills, Physical Activity, and Leisure Time Behaviour in Preschool-Aged Children | 10.3390/children9030431 | Exposure: Evaluates use of media (not specified), TV and computer. |
| Ni’na *et al.* | 2022 | Relationship between exposure to mobile phones and other electronic products and the ability development in children | 10.3760/cma.j.cn101070-20201003-01581 | Language: Chinese |
| Yu *et al.* | 2022 | High levels of screen time were associated with increased  probabilities of lagged development in 3-year-old children | 10.1111/apa.16373 | Exposure: Includes other devices in the analysis of other screens. |
| Takahashi *et al.* | 2023 | Screen Time at Age 1 Year and Communication and Problem-Solving Developmental Delay at 2 and 4 Years | 10.1001/jamapediatrics.2023.3057 | Exposure: Only evaluates screen time in general |
| Binet *et al.* | 2024 | Preschooler Screen Time During the Pandemic Is Prospectively Associated With Lower Achievement of Developmental Milestones | 10.1097/DBP.0000000000001263 | Outcome: Not assessed Motor development |
| Pekpak *et al.* | 2024 | Relationship Between Screen Time with Posture and Motor Coordination in Childhood: A Descriptive Study | 10.5336/pediatr.2024-101303 | Outside the age group  (6-14 years) |
| Ramesh *et al.* | 2024 | A cross sectional descriptive study on the effect of screen time on neurodevelopment, sleep, behavior and health of children aged 1-5 years | 10.70034/ijmedph.2024.3.38 | Descriptive study, does not analyze the association between devices and motor development |
| Yuan *et al.* | 2024 | The relationship between screen time and gross motor movement: A cross-sectional study of pre-school aged left-behind children in China | 10.1371/journal.pone.0296862 | Exposure: Only evaluates screen time in general |
